# Reactive oxygen species inhibits *Listeria monocytogenes* invasion into HepG2 epithelial cells

**DOI:** 10.1002/fsn3.615

**Published:** 2018-06-29

**Authors:** Guo‐wei Chen, Man Wu, Wu‐kang Liu, Man‐man Xie, Wei‐sheng Zhang, En‐guo Fan, Qing Liu

**Affiliations:** ^1^ School of Medical Instrument and Food Engineering University of Shanghai for Science and Technology Shanghai China; ^2^ Anorectal Department of Gansu Provincial Hospital Lanzhou China; ^3^ Institute of Biochemistry and Molecular Biology ZBMZ University of Freiburg Freiburg Germany

**Keywords:** HepG2 epithelial cells, inflammatory cytokines, Invasion, *Listeria monocytogenes*, reactive oxygen species

## Abstract

*Listeria monocytogenes* (Lm) can colonize human gastrointestinal tract and subsequently cross the intestinal barrier. Reactive oxygen species (ROS) are produced by NADPH oxidase. However, the role of ROS in bacterial invasion remains to be less understood. Herein, we investigated the impact of ROS on Lm invasion to HepG2 using NADPH oxidase inhibitor, diphenyleneiodonium chloride (DPI), as well as the ROS scavenger, *N*‐acetyl cysteine (NAC). Our results showed that inhibiting ROS increased the invasive capability of Lm. Moreover, after Lm infection, inflammatory cytokines such as tumor necrosis factor alpha (TNF‐α) and interleukin 1beta (IL‐1β) in HepG2 were significantly upregulated. However, after inhibiting ROS, the expression levels of TNF‐α and IL‐1β were downregulated, indicating a failure of host cells to activate the immune mechanism. Taken together, ROS in Lm might be as a signal for host cells to sense Lm invasion and then stimulate cells to activate the immune mechanism.

## 1 INTRODUCTION

*Listeria monocytogenes* (Lm) is a Gram‐positive and facultative intracellular pathogen of humans and animals. It is responsible for severe food‐borne infections primarily in immunocompromised individuals (Doganay, 2003; Farber & Peterkin, 1991; Mclauchlin, 1997). Serovars 1/2a, 1/2b, 1/2c, and 4b of Lm cause the majority of documented listeriosis (Swaminathan & Gerner‐Smidt, 2007). Listeria pathogenesis involves the ingestion of contaminated foods. Lm escapes the host immune system and penetrates the intestinal barrier via passive phagocytosis or active invasion of epithelial cells with subsequent hematogenous spread into the liver, spleen, and other organs (Velge & Roche, 2010). Several studies have characterized the manner by which Lm crosses the intestinal barrier and have identified important virulence factors that mediate its internalization, escape from the phagosome, and intra‐ and intercellular movements (Ireton, 2007; Pizarro‐Cerdá & Cossart, 2009). Several membrane proteins of Lm interact with abiotic and biotic surfaces and are essential for bacterial adhesion and invasion (Cabanes, Dussurget, Dehoux, & Cossart, 2004; Camejo et al., 2011; Cossart, 2011; Cossart, Pizarro‐Cerda, & Lecuit, 2003; Javier & Pascale, 2006; Mélanie, Bierne, & Cossart, 2006; Seveau, Pizarro‐Cerda, & Cossart, 2007; Stavru, Archambaud, & Cossart, 2011). Listeriolysin O is a multifunctional virulence factor that plays a role in bacterial proliferation and host mucosal cell extravasation (Ireton, 2007; Pizarro‐Cerdá & Cossart, 2009). The membrane protein ActA, encoded by the *actA* gene, is a bacterial aggregation factor that promotes Lm proliferation in host cells through excited protein molecule polymerization and also plays a role in bacterial internalization into host cells. Internalin and InlB are membrane proteins that facilitate the entry of Lm into (Decatur & Portnoy, 2000) nonphagocytic host cells (Decatur & Portnoy, 2000; Moors, Levitt, Youngman, & Portnoy, 1999; Shaynoor & Pascale, 2002). Limited studies on the molecular mechanism of Lm invasion have mainly focused on several known virulence factors, including PrfA, ActA, and SigmaB (Dramsi et al., 1995; Gaillard, Berche, Frehel, Gouln, & Cossart, 1991; Parida et al., 1998).

Nicotinamide adenine dinucleotide phosphate oxidase (NADPH oxidase, NOX), a well‐known reactive oxygen species (ROS) producer in animal and plant cells, is mainly composed of five subunits including gp91^phox^, p22^phox^, p47^phox^, p40^phox^, and p67^phox^ and activated by environmental stress to generate ROS that in turn modulates signal transduction and gene expression regulation (Luo et al., 2013; Schwab, Hu, Wiedmann, & Boor, 2005; Travier et al., 2013). NOX has been reported to exist in 19 bacteria (Lin‐Bo, Luo, Qu, Xiong, & Li, 2010). According to its biological function, it is divided into three groups, such as aerobic bacteria‐specific H_2_O_2_ NOX1 (H_2_O_2_‐forming NADH oxidase) (David & Mayhew, 1998), H_2_O NOX2 (H_2_O‐forming NADH oxidase) (Schmidt, Stöcklein, Danzer, Kirch, & Limbach, 1986), and NOX3 ultra‐oxygen anion(O_2_.^−^) (O_2_.^−^—forming NADH oxidase), respectively (Higuchi, Yamamoto, & Kamio, 2000). NOX research in bacteria has primarily focused on Streptococcus and lactic acid bacteria. Koike and colleagues (Koike, Kobayashi, Ito, & Saitoh, 1985) first discovered the activity of NOX in *Leuconostoc mesenteroides*. Patchett and colleagues (Patchett, Kelly, & Kroll, 1991) observed NOX activity when studying oxygen metabolism in NCTC 7973 (Lm strain). Researchers have characterized a series of NOX isoforms named gp91phox homolog, which includes NOX1, NOX2 (gp91phox), NOX3, NOX4, NOX5, DUOX1, and DUOX2 (Adolph, Schoeniger, Fuhrmann, & Schumann, 2012; Kuczyńska‐Wiśnik et al., 2010; Schillaci, Arizza, Dayton, Camarda, & Stefano, 2008). ROS generated by NADPH oxidase can trigger host cell apoptosis when stimulated by exogenous signals (Garner, James, Callahan, Wiedmann, & Boor, 2006). However, the role of ROS during pathogen invasion into host cells remains unknown. Only a few studies have evaluated the exact relationship between ROS and Lm invasion. Thus, we investigated the role of ROS during Lm invasion using DPI and NAC to induce ROS in Lm. HepG2 cells were used, and inflammatory cytokines, TNF‐α and IL‐1β, in cells were also detected.

## 2 MATERIALS AND METHODS

### 2.1 Bacterial strains and cell lines

Lm‐ATCC 43251 was provided by Shanghai Prajna Biology Technique Co., Ltd. (Shanghai, China), and Lm‐EGDe was donated by Associate Professor Luo Qin, Central China Normal University. The bacteria were cultured in liquid medium of brain–heart infusion (BHI) (Beijing Land Bridge Technology, Beijing, China) supplemented with 50% glycerol and 1.5% agar, and the cultures were stored at −80°C. The strain was incubated in 50 ml of BHI and shaken overnight under 37°C with 100 r/min shaking. A 1 ml aliquot of cultures was transferred into 50 ml of fresh BHI and shaken at 130 r/min and 37°C to obtain an optical density at 600 nm of 0.3 with a microplate reader (Molecular Devices, California, and USA).

Human hepatocytes (HepG2 cells, ATCC HB‐8065) were cultured in Dulbecco's modified Eagle's medium(DMEM, Gibco BRL) supplemented with 10% fetal bovine serum (Hyclone), 1 mmol/L sodium pyruvate, 100 U/ml penicillin, and 100 mg/ml streptomycin (Sigma) at 37°C in a 5% CO_2_ atmosphere. Cells were seeded in 12‐well tissue culture plates and grown for 24 hr (HepG2; 1 × 10^5^ cells/well) before infection.

### 2.2 Determination of Lm viability

Bacterial suspensions with an optical density at 600 nm of 0.3 were statically incubated with DPI of 0, 0.1, 0.5 1, 2 μmol/L at 37°C for 30 min. Then, the samples were centrifuged at 2,057 *g* for 10 min, washed with PBS for three times, and finally resuspended in fresh BHI. Aliquots of 200 μl samples were added per well, and absorbance was measured at 600 nm with a microplate reader. The viability of Lm was estimated by 3‐[4,5‐dimethylthiazol‐2‐yl]‐2,5‐diphenyltetrazolium bromide (MTT) (Beyotime Institute of Biotechnology, Shanghai, China) with some modifications recommended as per (Schillaci et al., 2008). 20 μl of 1 mg/ml MTT was added per well. The plates were placed statically and then incubated at 37°C for 4 hr. The supernatant was separated from the medium by centrifugation at 2,057 *g* for 10 min. Insoluble purple formazan was dissolved in 150 μl of DMSO. Bacterial viability was assessed by absorbance at OD_570 nm._

For treatment of Lm with NAC or DPI, the suspensions at an optical density of 0.3 at 600 nm were mixed with NAC or DPI at different concentrations (0, 0.1, 0.5, 1, 2 mmol/L) and incubated at 37°C for 14 hr under static condition. 200 μl samples were added per well, and absorbance at 600 nm was measured with a microplate reader. 20 μl of 1 mg/ml MTT was added per well.

### 2.3 ROS detection

Reactive oxygen species was measured using 2′,7′‐dichlorodihydrofluorescein dictate as probe (DCFH‐DA; Sigma, California, USA) as described by Dorota et al. (Kuczyńska‐Wiśnik et al., 2010). Briefly, 10 μmol/L DCFH–DA was added into each well and incubated with Lm or HepG2 cells at 37°C for 30 min. The level of fluorescence was detected using a microplate reader (SpectraMax M2, Molecular Devices, and USA) (excitation, 488 nm; emission, 530 nm).

### 2.4 Lm invasion assays

HepG2 cells in DMEM (Gibco) were inoculated with bacterial suspension (1 × 10^7^ CFU/ml) to obtain a multiplicity of infection of 1:100 for 1.5 hr at 37°C in the presence of 5% CO_2_. After infection, extracellular bacteria were abrogated by gentamicin (500 μg/ml) in PBS (0.01 mol/L, pH 7.2) for 1 hr. HepG2 cells were washed thrice with PBS to remove no adherent bacteria. Invasive bacteria were harvested after HepG2 lysis using a lysis solution (1% Triton X‐100; Sigma). The concentration of invasive bacteria was determined by measuring optical density of the bacterial suspension via plate counting. The experiment was performed in five independent experiments.

### 2.5 qPCR of TNF‐α and IL‐1β

After Lm invasion, the RNA of HepG2 cells was extracted using Trizol (TaKaRa, Japan). qPCR was performed using the PrimeScript™ First‐Strand cDNA Synthesis Kit (TaKaRa, Japan). The primer pairs shown in Table 1 were synthetized by Sangon Biotech (Shanghai, China), and SYBR GREEN real‐time quantitative PCR was performed with the ABI Prism 7900HT Sequence Detection System (Applied Biosystems, Foster City, CA). The relative quantification values of the genes were calculated using the comparative threshold cycle (△△CT) method.

**Table 1:** Primer sequences used in qRT‐PCR analysis

Primer name	Sequence
TNF‐α‐forward	TCCTTCAGACACCCTCAACC
TNF‐α‐reverse	ATCCCAGGTTTCGAAGTGGT
IL‐1β‐forward	TCAGCACCTCTCAAGCAGAA
IL‐1β‐reverse	TCCACATTCAGCACAGGACT
β‐Actin‐forward	ACTCTTCCAGCCTTCCTTCC
β‐Actin‐reverse	CGTACAGGTCTTTGCGGATG

### 2.6 Immunofluorescence staining and confocal microscopy image

HepG2 cells were grown on coverglass (Thermo Fisher Scientific, USA), cocultured with Lm, and then washed three times. Cells were then fixed with 1% formaldehyde in PBS at room temperature for 30 min and then permeabilized in 0.1% Triton X‐100 in PBS for 10 min. Cells were incubated at 37°C for 2 hr with anti‐Lm polyclonal antibody(Shanghai Prajna Biology Technique Co., Ltd. China). HepG2 cells were then incubated with Alexa Fluor 594 goat anti‐rabbit IgG (H+L) (Life Technologies, USA). Extensive washing in PBS, the cells were mounted on slides using a DAPI (H‐1200; VectorLab.) mounting medium. Images were obtained under confocal laser scanning microscope (Nikon, Tokyo, Japan).

### 2.7 Statistical analysis

All experiments in this study were performed in *n* = 5 independent experiments. Data analysis and graphical evaluations were conducted using GraphPad Prism 5.0 (GraphPad Software Inc., San Diego, CA).

## 3 RESULTS

### 3.1 DPI and NAC significantly downregulate ROS without affecting Lm proliferation and viability

**Figure 1:**
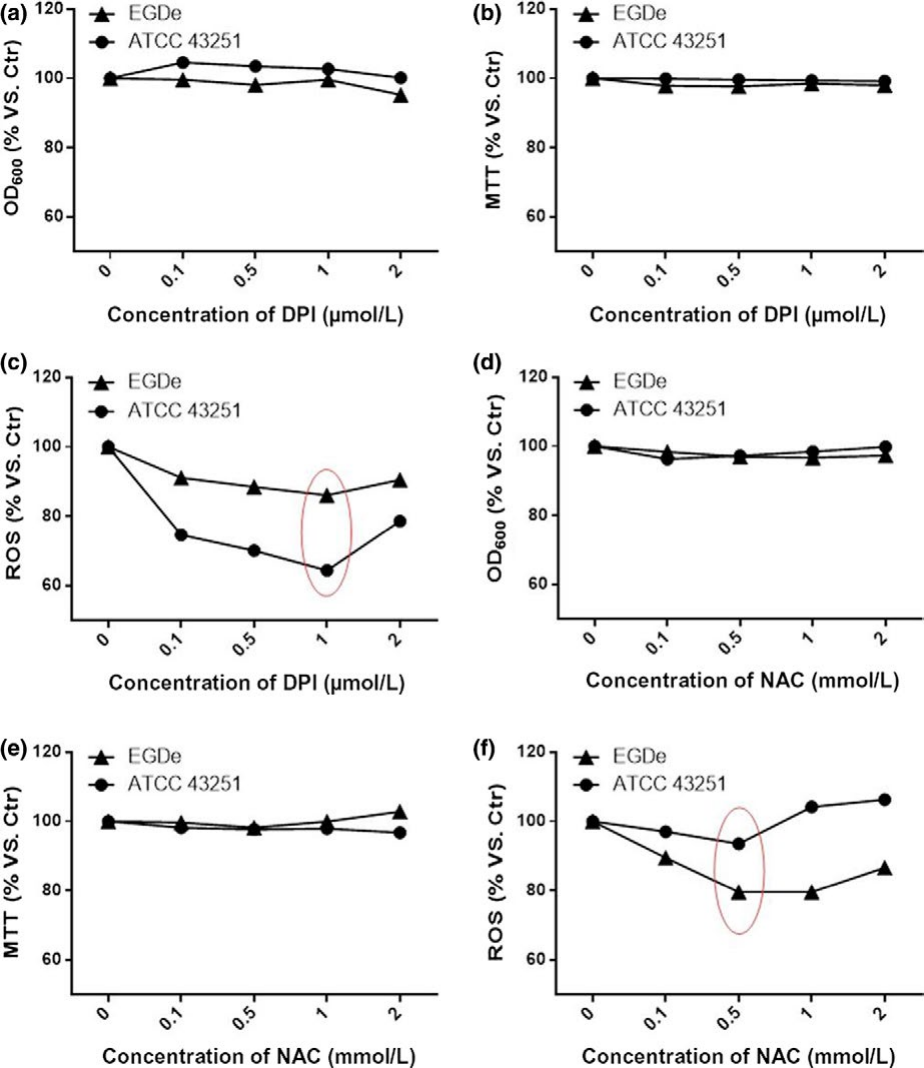
DPI and NAC can efficiently downregulate ROS level without affecting Lm viability. (a) Proliferation of DPI‐ or NAC‐treated Lm in BHI at 37°C; (b, e) viability of DPI‐ or NAC‐treated Lm in BHI at 37°C; (c, f) ROS production of DPI or NAC treatment. Data are mean ± SEM. BHI, brain–heart infusion; DPI, diphenyleneiodonium chloride Lm, *Listeria monocytogenes*; NAC, *N*‐acetyl cysteine; ROC, reactive oxygen species

To explore whether or not DPI and NAC affect the growth of Lm, EGDe and ATCC 43251 Lm strains were treated with different concentrations of DPI or NAC, and the optical density of the bacterial suspension was tested in 96‐well plates. Figure 1a and d shows that the OD_600 nm_ of wild strain EGDe and ATCC 43251 suspensions did not significantly differ among the different DPI or NAC concentrations. Lm viability was also tested with an MTT assay. As shown in Figure 1b and e, DPI or NAC exhibited no harm to Lm viability compared with untreated cells. From Figure 1c and f, both DPI and NAC can markedly suppress the ROS production of the two strains. And both strains act in the same fashion. The ROS production of ATCC 43251 was lower than EGDe in the five different DPI concentrations of DPI group (Figure 1c). However, the ROS production of ATCC 43251 was higher than EGDe in five different NAC concentrations of NAC group (Figure 1f). The results showed that ATCC 43251 and EGDe have different sensitivity to DPI or NAC. It seems ATCC 43251 is more sensitive to the DPI than NAC, and EGDe is more sensitive to NAC.

### 3.2 Lm invasion is enhanced following ROS suppression by DPI or NAC

**Figure 2:**
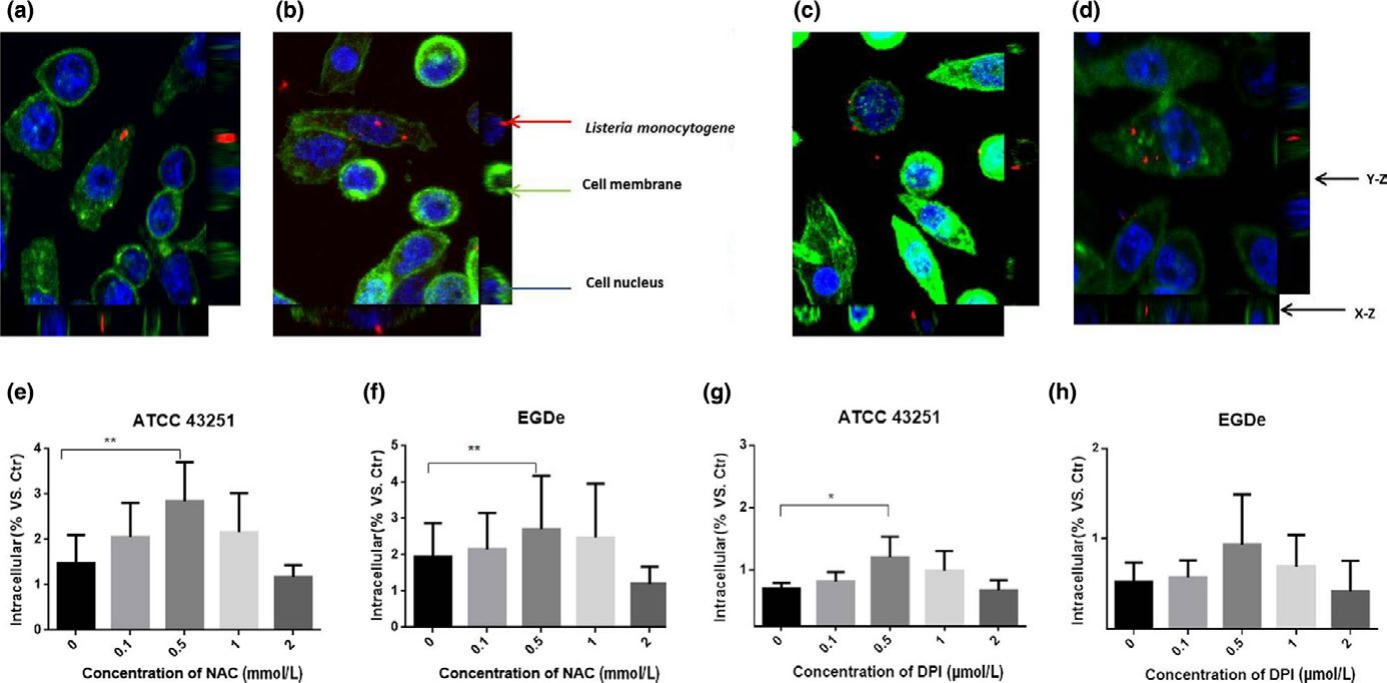
Invasion efficiency of Lm on HepG2 cells increased when ROS were inhibited by DPI or NAC. Confocal micrograph of 0 (a) or 1 (b) μmol/L DPI‐treated Lm (ATCC 43251); confocal micrograph of 0 (c) or 0.1 (d) mmol/L NAC‐treated EGDe; Lm, cell nucleus, and cytomembrane were stained as red, blue, and green, respectively, in this immunofluorescence assay. The invasiveness of NAC‐treated ATCC 43251 (e) or EGDe (f) and DPI‐treated ATCC 43251 (g) or EGDe (h) was estimated by plate counting. Data are mean ± SEM, *p < .05, **p < .01. DPI, diphenyleneiodonium chloride; Lm, *Listeria monocytogenes*; NAC, *N*‐acetyl cysteine; ROC, reactive oxygen species

In order to evaluate the role of Lm‐produced ROS in host invasion, Lm was treated with a series of concentrations of DPI and NAC prior to infection of HepG2 cells. As shown in Figure 2b and d, immunofluorescence staining of Lm with confocal imaging indicates that treatment by either 1 μmol/L DPI or 0.1 mmol/L NAC facilitates invasion into HepG2 cells as evidenced by the qualitatively increased presence of intracellular Lm (stained red). In comparison, untreated Lm could also invade HepG2 cells, but in a less efficient manner (Figure 2a,c). These results were verified in a quantitative manner by cell lysis plate counting assay as shown in Figure 2e–h. NAC treatment improved invasion efficiency at concentrations of 0.1 to 1 mmol/L for ATCC 43251 (Figure 2e) and EGDe (Figure 2f). This corresponded with a decrease in ROS levels, especially for wild strain EGDe, as shown in Figure 1f. In addition, for DPI treatment the optimal invasion efficiency was observed at concentrations of 0.1 to 1 μmol/L for ATCC 43251 (Figure 2g) and 0.5 and 1 μmol/L for EGDe (Figure 2h) strains, and corresponding ROS suppression as shown in Figure 1c. The invasion efficiency was lowest for 2 mmol/L NAC or 2 μm DPI‐treated Lm which correlated with the highest ROS levels as shown in Figure 1c,f. These findings show the suppression of Lm ROS production promotes host invasion.

### 3.3 Influence of Lm invasion on the ROS production of HepG2 cells

**Figure 3:**
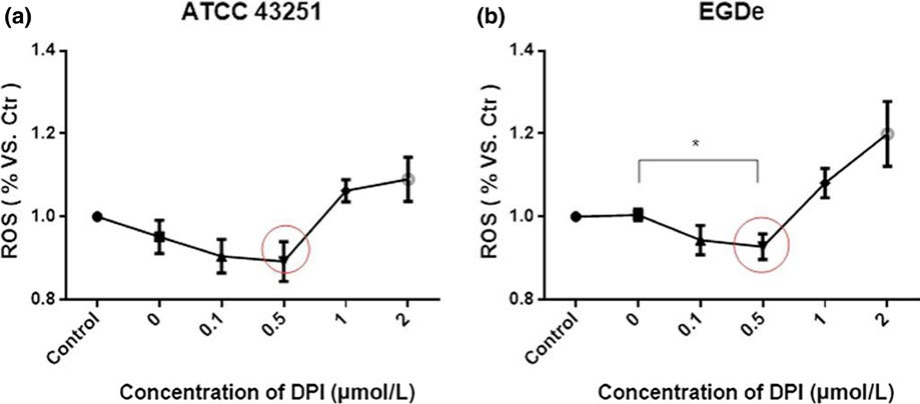
ROS production of HepG2 cells after invasion by DPI‐treated Lm 43251(A) or EGDe. Data are mean ± SEM, *p < .05. DPI, diphenyleneiodonium chloride; Lm, *Listeria monocytogenes*; ROC, reactive oxygen species

Reactive oxygen species production by HepG2 cells was evaluated to explore the changes after Lm invasion. HepG2 cells infected with 0.5 μmol/L DPI‐treated EGDe showed the lowest ROS production than cells infected with untreated EGDe (Figure 3b). The same tendency was showed between Lm ROS and HepG2 cells, and ROS levels were also lower in DPI‐treated Lm compared to NAC‐treated Lm. (Figure 1c and f). Although the relationship between Lm and HepG2 ROS levels is unclear from these experiments, our results do suggest the important role of ROS in Lm invasion.

### 3.4 Influence of invasiveness on the inflammatory cytokines of HepG2 cells

**Figure 4:**
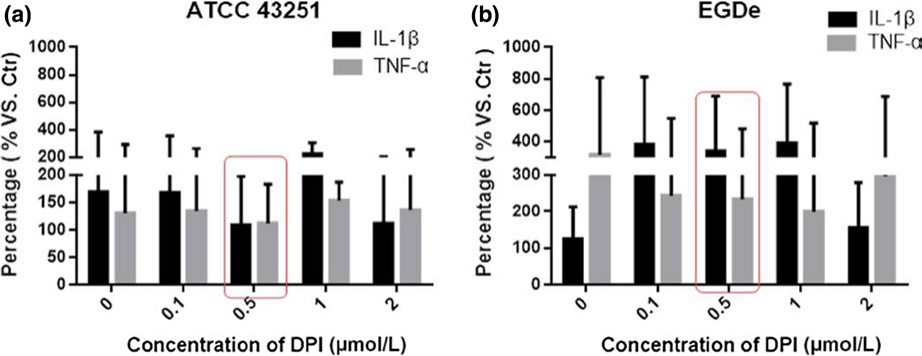
Influence of invasiveness on the inflammatory cytokines of HepG2 cells. Expression levels of TNF‐α and IL‐1β after the HepG2 cells were invaded by Lm 43251(a) or EGDe (b). Data are mean ± SEM. Lm, *Listeria monocytogenes*

TNF‐α and IL‐1β are proinflammatory cytokines released by infiltrated leukocytes in the setting of microbial infection. In HepG2 with infection of ATCC 43251 + 0.5 μmol/L DPI treatment, the expression of TNF‐α is decreased about 26.7% and the expression of IL‐1β is increased about 29.4% comparing with that of without DPI treatment (Figure 4a). While for cells with EGDe infection, the alterations of inflammatory cytokines expression were larger with 42.9% TNF‐α reduction and 233.3% IL‐1β increase by 0.5 μmol/L DPI treatment relative to 0 μmol/L DPI treatment (Figure 4b). These data reveal inflammatory cytokines upregulated during Lm invasion may be modulated by ROS.

## 4 DISCUSSION

In this study, we employed DPI and NAC to suppress ROS levels in Lm in order to explore the role of ROS in invasion. Our findings indicate that ROS suppression in Lm‐ATCC 43251 with NAC could promote invasion and suppress proinflammatory mediators such as TNF‐α. When ROS suppression in Lm with DPI in 0.5 and 1 μmol/L, the invasion ability was increased too. This novel finding suggests that ROS production by Lm plays an important role during invasion process. We speculate that ROS production by Lm can be detected by host cells and trigger the immune system under normal circumstances. Upon ROS suppression by DPI or NAC, host cell detection of Lm infection is inhibited with a muted immune system response resulting in enhanced Lm infection. These preliminary findings provide an intriguing mechanism for mediating successful Lm invasion. NOX is a well‐known ROS producer in animal and plant cells that is mainly composed of five subunits gp91^phox^, p22^phox^, p47^phox^, p40^phox^, and p67^phox^. Researchers have characterized a series of NOX isoforms named gp91phox homolog, which includes NOX1, NOX2 (gp91phox), NOX3, NOX4, NOX5, DUOX1, and DUOX2{Moors, 1999 #18}{Parida, 1998 #19}{Gaillard, 1991 #20}. It can be activated by environmental stress to generate ROS that in turn modulates signal transduction and gene expression regulation{Dramsi, 1995 #21}{Luo, 2013 #22}{Schwab, 2005 #23}. Further study will be continued, and we will research the invasion ability of NOX deletion EGDe. Other questions require future investigation, including which enzyme in Lm is responsible for ROS production, and what differences exist between bacteria NOX and NADPH oxidase in terms of its formation and function.

Our findings also raise questions regarding the use of antioxidants in over‐the‐counter medications and supplements. As this study suggests ROS enhances Lm invasion, evaluating whether antioxidants promote an increased risk of certain microbial infections warrants future investigation.

## CONFLICT OF INTEREST

We confirm that there is no conflict of interest exists for this manuscript.
